# Enhancing the general public knowledge of antimicrobial resistance (AMR) in Africa: a video-based Brief Educational Resource Review

**DOI:** 10.1093/jacamr/dlaf005

**Published:** 2025-01-29

**Authors:** Jimmy Nkaiwuatei, Hafeez Hamza, Samar Akbi, Ngoni Muzondo

**Affiliations:** Research Department, Zihi Institute, Nairobi, Kenya; Research Department, Zihi Institute, Nairobi, Kenya; Research Department, Zihi Institute, Nairobi, Kenya; Faculty of Pharmacy, University of Algiers 1, Algiers, Algeria; Research Department, Zihi Institute, Nairobi, Kenya; Harare Institute of Technology, Harare, Zimbabwe

## Abstract

**Background:**

The ability of microorganisms to resist antimicrobial medicines is called antimicrobial resistance (AMR). AMR awareness among the general public may be increased via a variety of approaches including the use of social media campaigns, traditional media, influencer outreach storytelling, community theatre, interactive games and quizzes and art and music. This analysis aims to evaluate YouTube videos to educate the general public about AMR.

**Methods:**

A content analysis was performed on the AMR educational videos that were retrieved from YouTube using the following keyword phrases: ‘what is antimicrobial resistance’, ‘causes of antimicrobial resistance’ and ‘prevention of antimicrobial resistance’.

**Results:**

A total of 74 AMR educational videos were identified and analysed based on predefined selection criteria. Subsequently, three online videos that met the inclusion criteria were selected and analysed.

**Conclusions:**

The analysed AMR educational videos were easily accessible and comprehensible, and serve as valuable resources for promoting AMR awareness and education among the general population.

## Introduction

Antimicrobial resistance (AMR) occurs when the antimicrobial drugs are no longer able to treat infections.^1^ Lack of knowledge and understanding of AMR is one of the factors attributed to its growth and subsequent transmission.^2^ Studies have shown that the general public in low-income countries has limited knowledge and awareness of AMR.^3^ A systematic review of 77 studies across 24 low- and middle-income countries from Africa and Asia found that only 34.5% of the general public had adequate knowledge about AMR.^4^

The level of public knowledge about AMR in middle-income countries is slightly better compared with low-income countries, but still suboptimal.^5^ A survey across five middle-income countries (Brazil, China, India, Russia and South Africa) found that only 36% of respondents were aware of the term ‘antibiotic resistance’,^6^ while the general public in high-income countries tends to have better knowledge and awareness of AMR than those in low- and middle-income countries.^7^ A survey in the USA found that 73% of the public knew antibiotic resistance was a major public health problem.^8^ Another study in the UK reported that 81% of the public understood that overuse of antibiotics can lead to AMR.^9^

Increased awareness of AMR can positively influence antimicrobial stewardship attitudes and approaches. Studies show that healthcare professionals, when exposed to targeted education about AMR, demonstrate better stewardship practices, such as prescribing antibiotics more judiciously and adhering to guidelines.^10^ Training programmes often lead to enhanced knowledge about the risks of overprescribing and the importance of preserving antibiotic efficacy.

Countries with greater awareness of AMR and robust AMR stewardship programmes often report better control over the spread of MDR infections.^10^ For instance, countries with comprehensive public health campaigns, better surveillance systems and well-implemented stewardship programmes, such as those in Northern Europe (e.g. Sweden, Norway and Finland), tend to have lower rates of antibiotic resistance compared with countries with less emphasis on AMR control.^11^

AMR has been associated with lack of effective communication due to the use of complex terminologies, among other factors.^12^ Consequently, this has made it difficult for the general public to clearly comprehend this health problem. Therefore, this study aims to explore AMR-focused educational YouTube videos as a strategy to promote AMR knowledge and understanding among school-aged children and young adults. Studies indicate that YouTube has emerged as an efficient and reliable medical educational resource because of its ease of access and use, coupled with the increase in online presence, especially by school-aged children and young adults.^13^

The objectives of this review were: (i) to generate evidence on the availability and accessibility of AMR-focused educational YouTube videos to school-aged children and young adults in Africa; (ii) to assess the simplicity of the language used in online AMR educational videos, with a focus on content that is suitable for non-expert audiences, such as school-aged children and young adults in Africa; (iii) to critically evaluate the quality and effectiveness of AMR-focused YouTube videos in raising awareness and promoting proper antimicrobial stewardship among school-aged children and young adults in Africa; and (iv) to explore AMR-focused educational YouTube videos as a strategy to promote AMR knowledge and understanding among school-aged children and young adults in Africa.

## Materials and methods

The videos used in this analysis were retrieved only from YouTube using the following keyword phrases; ‘what is antimicrobial resistance’, ‘causes of antimicrobial resistance’, and ‘prevention of antimicrobial resistance’. The reason why YouTube was selected as the only information resource for this study is because it has emerged as a highly popular and widely used online educational resource in medical education.^13^

**Figure 1.**
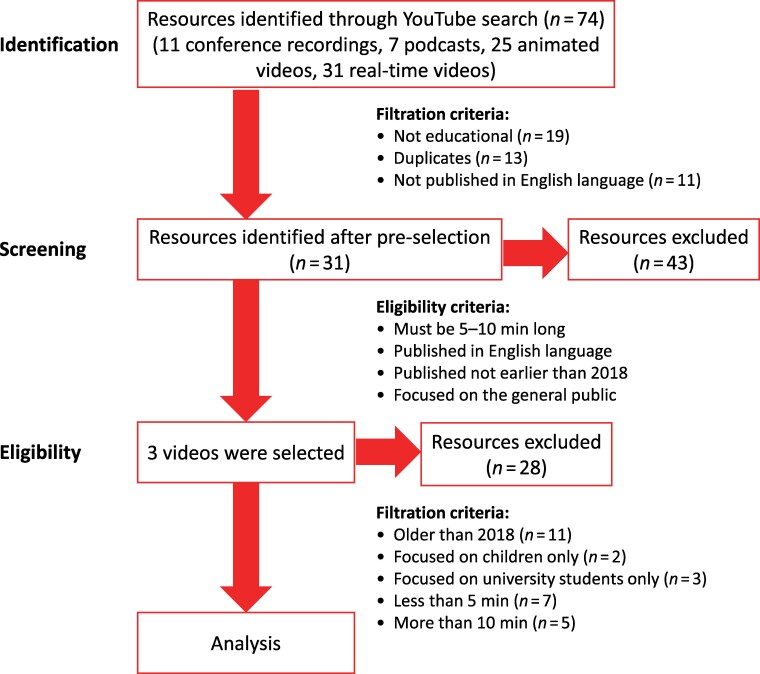
A schematic representation for the identification of study materials.

This search process was conducted between late October and early November 2023 on different servers from three different countries; Kenya, Zimbabwe and Algeria. Based on this search, a total of 74 diverse types of videos were obtained, including: 11 conference recordings; 7 podcast recordings; 25 animated videos (created with the aid of computer software/tools); and 31 real-time (created through live recordings using real cameras) videos, as shown in Figure 1. Out of these, 43 videos were filtered out due to duplication, not being educational and not being in English. These were further screened based on the following criteria that the resources must be: focused on the general public and be between 5–10 min long for effective engagement; must have not been published earlier than 2018 to represent current AMR knowledge and perspectives; produced in the English language as it is the most popular and widely used language for instructions in both formal and informal education in Africa;^14^ and must be educational (entail introduction to AMR and antimicrobials, as well as causes and prevention of AMR) for the benefit of the wide range of audience with varied levels of AMR understanding.

## Results and discussion

**Figure 2.**
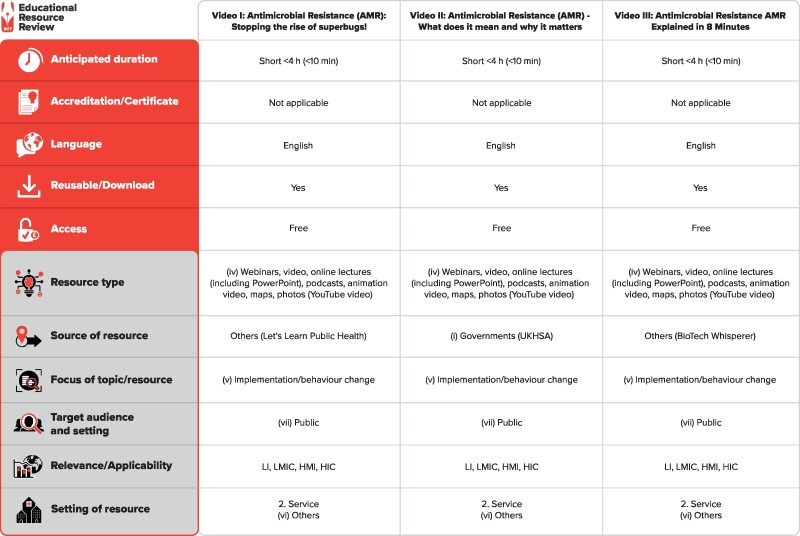
Characteristics of the analysed videos. Video 1: Antimicrobial resistance (AMR): stopping the rise of superbugs! Resource web link: https://www.youtube.com/watch?v=HZ4udzNJbrs. Video 2: Antimicrobial resistance (AMR)—what does it mean and why it matters. Resource web link: https://www.youtube.com/watch?v=MENdrA8B0N4. Video 3: Antimicrobial resistance (AMR) explained in 8 minutes. Resource web link: https://www.youtube.com/watch?v=FcjpbXD1wOA. The full classification scheme is available at http://bsac.org.uk/wp-content/uploads/2019/03/Educational-resource-review-classification-scheme.pdf. LI, low-income countries; LMIC, low- and middle-income countries; HMI, high- and middle-income countries; HIC, high-income countries.

Based on this analysis, one real-time and two animated videos were finally selected for analysis. These were: (i) ‘Antimicrobial resistance (AMR): stopping the rise of superbugs!’, published in 2022 by Let’s Learn Public Health; (ii) ‘Antimicrobial resistance (AMR)—what does it mean and why it matters’, published in 2021 by the UK Health Security Agency; and (iii) ‘Antimicrobial resistance (AMR) explained in 8 minutes’ published in 2022 by BioTech Whisperer (Figure 2). The quality of these videos was evaluated by assessing the depth and relevance of the content to the intended objectives. We also used a quality assessment tool called the global quality scoring (GQS) system, an international scoring system that is used to evaluate the quality of the information in YouTube videos on a 5-point scale.^15^

In one of the videos selected, ‘Antimicrobial resistance (AMR): stopping the rise of superbugs!’ James Clark narrates the importance of AMR prevention to ensure public health security. Like in the other two selected resources, this video gives a comprehensive educational narrative about antimicrobials and AMR, including its occurrence, spread and prevention. The video clearly explains how AMR occurs in bacteria, viruses, parasites and fungi, as well as the significant drivers such as misuse of antimicrobials in both humans and animals. It further informs viewers that when AMR occurs, it spreads across pathogens. Similar to the other two resources, this video also underscores the need for a One Health approach to AMR prevention. Based on this video, AMR can significantly compromise critical medical procedures such as surgery, chemotherapy and transplantation.

Interestingly, this video further highlights the economic impacts of AMR and how it can potentially impact all sectors both at an individual and organizational/system level. This is vital as it reinforces the importance of a multisectoral approach to combat it. The AMR language used in this video was simple to understand, and hence appropriate to the target audience.

In the second video, ‘Antimicrobial resistance (AMR)—what does it mean and why it matters’, Prof. Diane Ashiru-Oredope gives an enlightening introduction to microbes, antimicrobials and AMR. She narrates how AMR emerges in microbes and the practical ways to mitigate it. In this video, Prof. Ashiru-Oredope states that microbes are everywhere including in soil and air, and the majority of them play beneficial roles such as digestion, fighting pathogens and contributing to the ecosystem, while some are harmful. This resource was found to be suitable and effective in informing the non-expert AMR audiences about the roles of microbes in the environment and how to co-exist with them.

Based on this video, when the harmful microbes develop resistance to antibiotics, it becomes difficult to treat them, causing death. In the video, Prof. Ashiru-Oredope advises that behaviour change is key in fighting AMR. Similar to the other videos, the authors found this resource very educative to the non-expert AMR audience such as school-aged children and young adults in Africa.

Moreover, the third video, ‘Antimicrobial resistance (AMR) explained in 8 minutes’, offers a detailed exploration of the complexities surrounding AMR. With clear insights into its definition, prevalence, causes and impact on healthcare and resources, the video underscores the importance of drawing lessons from past pandemics such as COVID-19 in addressing and mitigating AMR. It effectively delves into the multifaceted perspectives of AMR, spanning medical, public health, financial and societal dimensions. As described in the first video, ‘Antimicrobial resistance (AMR): stopping the rise of superbugs!’, this video also emphasizes the importance of the One Health approach as an effective strategy to engage and educate both professionals and the general public about the consequences of AMR and the dire need to combat it. Furthermore, the video emphasizes the significance of advancements in research, discovery and infection prevention and control programmes as crucial tools to fight AMR. The quality of the videos in terms of clarity and depth of the AMR content covered was found to be satisfactory.

### Characteristics of the videos

#### Simplicity of the language

All the videos selected employed non-complex English language terminologies that are easily understandable by the general public, thus making them effective resources for creating AMR awareness among a wider audience.

#### Accessibility and availability

The videos are openly accessible and can be easily obtained using the search words mentioned in the Materials and methods section.

#### Effectiveness

They capture all the important components of an AMR educational video: definition, causes and prevention, coupled with their engaging presentations, making them effective resources for AMR education. This is appropriate for audiences with limited AMR knowledge such as school-aged children and young adults. Future authors may consider translating their videos into their respective local languages for further understanding by populations with limited English language understanding.

### Quality assessment

Quality analysis of the videos was performed using the GQS system shown in Table 1. This international scoring system is used to evaluate the quality of the information in YouTube videos on a 5-point scale.^15,16^ As shown in Table 2, Videos 1, 2 and 3 were given an average score of 4, 4.5 and 3, respectively, based on the following parameters: relevance of the information to the target audience; AMR concepts covered in the videos as per the selection criteria; and quality and clarity of the content.

**Table 1.** GQS criteria used to score YouTube videos containing information on AMR

Score	Global score description
1	Poor quality, poor flow of the site, most information missing, not at all useful for patients.
2	Generally poor quality and poor flow, some information listed but many important topics missing, of limited use to patients.
3	Moderate quality, suboptimal flow, some important information is adequately discussed, but others poorly discussed, somewhat useful for patients.
4	Good quality and generally good flow, most of the relevant information is listed, but some topics are not covered, useful for patients.
5	Excellent quality and excellent flow, very useful for patients.

**Table 2.** Table showing reviewers’ GQS-based scores for the three selected videos

Name of video	Reviewer 1 score	Reviewer 2 score
1 ‘Antimicrobial resistance (AMR): stopping the rise of superbugs!’, published in 2022 by Let’s Learn Public Health	4	4
2 ‘Antimicrobial resistance (AMR)—what does it mean and why it matters’, published in 2021 by the UK Health Security Agency	4	5
3 ‘Antimicrobial resistance (AMR) explained in 8 minutes’, published in 2022 by BioTech Whisperer	3	3

The videos provide very informative content on microbes, antimicrobials and AMR, in a manner that is easily understandable by a very wide audience. The inclusion of high-quality visuals, clear audio and captivating presentations in the videos contributes to effective communication of the content. Additionally, the videos encourage action by suggesting practical steps to reduce AMR, making the information not only informative but also actionable for viewers.

### Clarity of the videos and contents

The videos appear to be very clear and well organized, with a logical flow of information. They use simple language and provide examples to enhance clarity.

Further, the videos feature well-crafted audiovisuals and eye-catching presentations, ensuring that the school-aged children and young adults can easily follow and engage with the content.

### Recommendations

To support future efforts in this field, we propose the development of a centralized, accessible and open-access database that aggregates key AMR educational videos. Such a repository would provide a go-to platform for the general public, ensuring that the most effective and accurate resources are readily available. To make the database accessible at a global level, it would comprise globally representative data written in the English language with provision for conversion into other official United Nations (UN) languages.
Additionally, while English is a widely used language of instruction internationally, it is important to take into account AMR educational resources produced in other widely spoken languages, such as French and German, to improve AMR education and understanding. Calvo *et al.*^17^ found that a lack of understanding of AMR and the individual responsibilities to combat it is one of the hindrances in AMR mitigation.

### Conclusions

In summary, it is evident that YouTube hosts vast AMR content in terms of videos, which is easily accessible and understandable due to the simplicity of the English language used. This makes it an efficient educational resource for the general public, especially school-aged children and young adults in Africa due to their high presence online. The first video, ‘Antimicrobial resistance (AMR): stopping the rise of superbugs!’, gives a comprehensive introduction to AMR and how it occurs in viruses, fungi and bacteria. The second video, ‘Antimicrobial resistance (AMR)—what does it mean and why it matters’, emphasizes that microbes are found everywhere in the environment, and they can either be beneficial and harmful, thus humans should learn how to co-exist with them, while in the third video, ‘Antimicrobial resistance (AMR) explained in 8 minutes’, the author describes AMR as a complex and multifaceted challenge that requires a One Health approach to combat it. Going forward, local organizations in Africa should embrace using YouTube as an additional educational channel to disseminate AMR education to the general public. This, coupled with written information, will reinforce AMR understanding, especially among the non-expert AMR populations in Africa.
